# MRI-Based Radiomics for Preoperative Prediction of Lymphovascular Invasion in Patients With Invasive Breast Cancer

**DOI:** 10.3389/fonc.2022.876624

**Published:** 2022-06-06

**Authors:** Mayidili Nijiati, Diliaremu Aihaiti, Aisikaerjiang Huojia, Abudukeyoumujiang Abulizi, Sailidan Mutailifu, Nueramina Rouzi, Guozhao Dai, Patiman Maimaiti

**Affiliations:** Department of Radiology, The First People’s Hospital of Kashgar, Xinjiang, China

**Keywords:** breast cancer, lymphovascular invasion, magnetic resonance imaging (MRI), machine learning, radiomics

## Abstract

**Objective:**

Preoperative identification of lymphovascular invasion (LVI) in patients with invasive breast cancer is challenging due to absence of reliable biomarkers or tools in clinical settings. We aimed to establish and validate multiparametric magnetic resonance imaging (MRI)-based radiomic models to predict the risk of lymphovascular invasion (LVI) in patients with invasive breast cancer.

**Methods:**

This retrospective study included a total of 175 patients with confirmed invasive breast cancer who had known LVI status and preoperative MRI from two tertiary centers. The patients from center 1 was randomly divided into a training set (n=99) and a validation set (n = 26), while the patients from center 2 was used as a test set (n=50). A total of 1409 radiomic features were extracted from the T2-weighted imaging (T2WI), dynamic contrast-enhanced (DCE) imaging, diffusion-weighted imaging (DWI), and apparent diffusion coefficient (ADC), respectively. A three-step feature selection including SelectKBest, interclass correlation coefficients (ICC), and least absolute shrinkage and selection operator (LASSO) was performed to identify the features most associated with LVI. Subsequently, a Support Vector Machine (SVM) classifier was trained to develop single-layer radiomic models and fusion radiomic models. Model performance was evaluated and compared by the area under the curve (AUC), sensitivity, and specificity.

**Results:**

Based on one feature of wavelet-HLH_gldm_GrayLevelVariance, the ADC radiomic model achieved an AUC of 0.87 (95% confidence interval [CI]: 0.80–0.94) in the training set, 0.87 (0.70-1.00) in the validation set, and 0.77 (95%CI: 0.64-0.86) in the test set. However, the combination of radiomic features derived from other MR sequences failed to yield incremental value.

**Conclusions:**

ADC-based radiomic model demonstrated a favorable performance in predicting LVI prior to surgery in patients with invasive breast cancer. Such model holds the potential for improving clinical decision-making regarding treatment for breast cancer.

## Introduction

According to the 2018 global cancer statistics, breast cancer ranks second in the incidence of new cancers (approximately 11.6%) and fifth in cancer-related mortality (approximately 6.6%) (1). Breast cancer has an incidence rate of 24.2% and a mortality rate of 15%, making it the most malignant cancer among women and a veritable “killer of women” (1). The number of new cases of breast cancer has been increasing annually (2). Recent studies have shown that lymphovascular invasion (LVI) by tumors is a crucial prognostic factor affecting patient outcomes and clinical treatment options (3–5). The main causes of death among patients with breast cancer are cancer recurrence and metastasis. Lymphovascular metastasis is the most common form of metastasis in breast cancer and consists in the invasion of regional lymph nodes, allowing cancer cells to reach distant organs (6). In breast cancer, LVI can occur before the appearance of lymph node metastasis and is an indicator of poor prognosis (6). Thus, LVI is considered one of the major criteria for tumor staging, prognostic prediction, and the selection of treatment options (7–9). However, postoperative pathology is currently the only available tool to confirm that tumor vessels promote lymphatic and blood vessel growth and invasion, with no effective method for non-invasively predicting LVI status before surgery.

The emergence of radiomics brings new opportunities in this regard to the field of oncology (10). Radiomics refers to the high-throughput analysis of digitized quantitative and high-dimensional imaging data and integrates histopathology, machine learning, medical statistics, and computer science at multiple levels and from multiple perspectives to yield high-fidelity data for the comprehensive evaluation of various tumor phenotypes (11). Three previous studies applied MRI-based radiomics to predict LVI in patients with breast cancer (12–14); however their results were controversial. Liu et al. built a combined model incorporating dynamic contrast-enhanced (DCE)-based radiomics signature and MRI-reported axillary lymph node (ALN) status with an area under the curve (AUC) of 0.763 (12). Kayadibi et al. identified the apparent diffusion coefficient (ADC)-based radiomic signature as the best model, with an AUC of 0.732 (13). Zhang et al. found that fusion radiomic model of the T2-weighted imaging (T2WI), contrast-enhanced T1-weighted imaging (DCE), and ADC maps achieved better predictive efficacy for LVI status than either of them alone (14). In this current study, we aimed to develop and validate machine learning-based radiomic models using preoperative MRI images as a non-invasive tool for the prediction of LVI status in patients with invasive breast cancer.

## Materials and Methods

### Patient Population

This study was conducted in accordance with the Declaration of Helsinki (as revised in 2013). This retrospective cohort study was approved by institutional ethics board and informed consent was waived.

**Figure 1:**
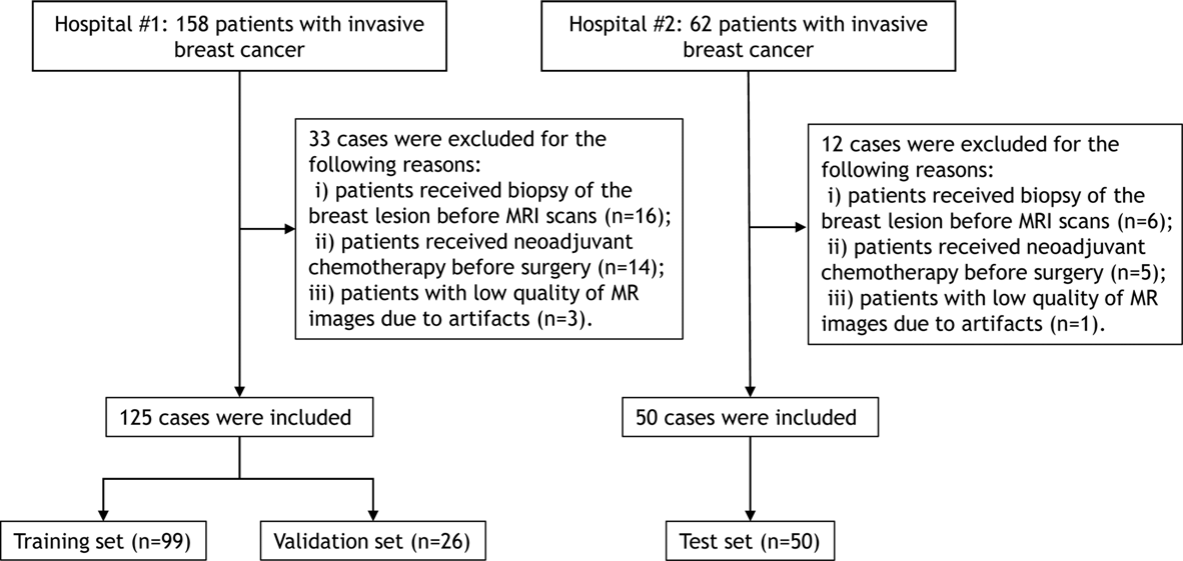
Flowchart for selection of the study population.

This study included a total of 175 consecutive patients with pathologically confirmed invasive breast cancer who underwent pretherapy contrast-enhanced MRI in two tertiary hospitals between January 2019 and October 2020. All eligible patients met the following inclusion criteria: i) patients with visible primary breast lesions on MRI, ii) patients with newly diagnosed invasive breast cancer by histopathological evaluation of a surgical specimens, and iii) patients underwent mastectomy or lumpectomy within two weeks after MRI scans. The exclusion criteria were as follows: i) patients received biopsy of the breast lesion before MRI scans, ii) patients received neoadjuvant chemotherapy before surgery, and iii) patients with low quality of MR images due to artifacts. The patient inclusion flowchart is shown in **Figure 1**.

The clinical and radiological information were included as follows: age, tumor location, tumor number, mass shape, tumor diameter, TNM stage, pathological ALN-status, internal enhancement pattern, background parenchymal enhancement, fibroglandular tissue, chest wall invasion, and pectoralis major muscle invasion. The assessment of radiological findings was in accordance with the American College of Radiology Breast Imaging Reporting and Data System (ACR BI-RADS) (15). Histological analysis was performed on specimens obtained at the surgery. Pathological ALN status was defined as positive if macrometastases or micrometastases were identified in one or more ALNs (12). LVI was assessed on hematoxylin and eosin-stained sections, and was defined as carcinoma cells in a definite endothelial-lined space in the peritumoral breast surrounding the invasive carcinoma (12). The specimens were analyzed by two pathologists with 5 and 16 years of experience in breast cancer who were blinded to the MRI findings.

### MRI Examination

All patients underwent conventional MRI, DCE-MRI, and diffusion-weighted imaging (DWI). A Siemens Avanto 1.5T superconducting magnetic resonance scanner, equipped with a 4-channel breast coil and a 6-channel body matrix coil, was used for MRI examination. A GE Signa HDxt 3.0T MR scanner, with an 8-channel phased-array breast coil, was used. Patients were placed in the prone position in the scanner, with both breasts hanging naturally in the coil. Axial and sagittal MR images were obtained from the axilla to the inferior margin of the breast, using the following imaging sequences and parameters: LAVA DCE sequence (repetition time [TR], 5.68 ms; time to echo [TE], 2.20 ms; inversion time [TI], 16 ms; slice thickness, 2.0 mm; field of view [FOV], 340 mm × 340 mm; and matrix, 348 × 348) and STIR T2WI sequence (TR, 11000 ms; TI, 240 ms; TE, 60 ms; slice thickness, 4.0 mm; slice interval, 0.4 mm; FOV, 340 mm × 340 mm; matrix, 320 × 192). DWI was performed using a single-shot SE-EPI sequence, with the following parameters: b, 800 s/mm^2^; TR, 6600 ms; TE, 60 ms; slice thickness, 4.0 mm; slice interval, 0.4 mm; FOV, 340 mm × 349 mm; and matrix, 130 × 96. For enhanced imaging, a double-barreled high-pressure syringe was used to inject the contrast agent, gadolinium-diethylenediamine-pentaacetic acid (DPTA), at a flow rate of 2.5 mL/s and a dose of 0.1 mmol/kg. A repeat LAVA DCE was obtained after administration of the contrast agent, using the same parameters as for the plain sequence. Each phase of imaging was 60s in duration, with eight phases completed, for 480s of imaging.

### Imaging Preprocessing

Given that the MRI images were acquired from different machines with different parameters, it is needed to eliminate the internal dependence of radiomic features on voxel size. Thus, we used the resampling method with linear interpolation algorithm to normalize the voxel size.

### Lesion Delineation and Segmentation

All patients’ T2WI, DCE, DWI, and ADC maps were exported from the picture archiving and communication system into the Radcloud (Huiying Medical Technology Co.,Ltd, Beijing, China) software. Subsequently, the region of interest (ROI) was manually and volumetrically segmented by a radiologist with 5 years of experience. All ROIs were then reviewed by a radiologist with 10 years of experience.

**Figure 2:**
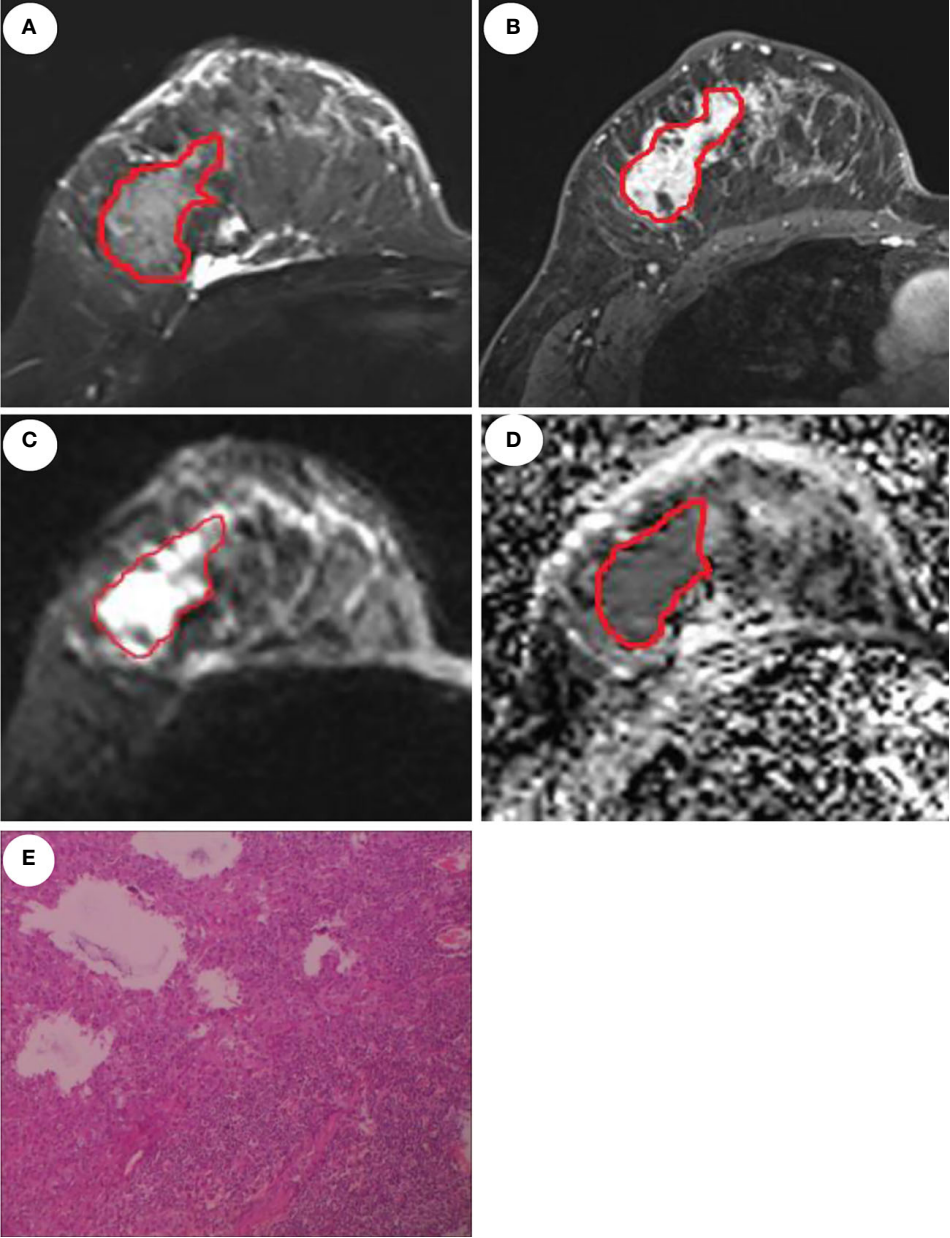
MRI and pathological images of a 46-year-old patient with invasive ductal carcinoma and LVI. Manual delineation of the region of interest on the T2WI **(A)**, DCE **(B)**, DWI **(C)**, and ADC **(D)**, respectively. **(E)** displays the presence of LVI.

ROI delineation rules were as follows: first, on the DCE images, the phase I image with the highest intensity was selected and an ROI was contoured along the margin of the tumor. Second, on the T2WI image, the primary tumor was defined by contouring the margin of the tumor which had a slightly higher signal. Third, the ROI on the DWI images was draw to cover the entire high-signal-intensity area. Finally, the ROI on DWI images was then transferred to ADC maps. The ROI excluded any visible liquefaction, necrosis, and cystic regions. **Figure 2** shows the ROI delineation and pathological image of a case with presence of LVI.

### Feature Extraction

A total of 1409 radiomic features were extracted from the DCE, T2WI, DWI, and ADC maps using the Pyradiomics function package (https://pyradiomics.readthedocs.io/). These features were divided into the following three categories: i) First-order statistical features (n=19), such as peak value, mean, variance, quantitatively describe the voxel intensity distribution of the lesion area in MR images through common basic indicators; ii) Shape-based features (n=16), describe the shape and size of the lesion; iii) Texture features (n=72), including Gray Level Co-occurrence Matrix (GLCM, n=24), Gray Level Dependence Matrix (n=16), Gray Level Run Length Matrix (GLRLM, n=16), and Gray Level Size Zone Matrix (GLSZM, n=16); and iv) and (iv) filter-derived features (n=1302): filter ‘wavelet’: n = 744; other filter (‘lbp’, ‘square’,’squareroot’, ‘logarithm’, ‘exponential’, ‘gradient’): n = 93 ×6 = 558. The detailed calculation formula for each radiomic feature is provided on the official website (https://pyradiomics.readthedocs.io).

### Feature Selection and Radiomic Model Construction

Before the selection of radiomic feature, the normalization processing of all extracted features was performed. To verify the credibility of the manual segmentation between the two radiologists, the MRI scans of 30 patients were randomly selected and segmented by the two radiologists for double-blind interpretation. Interclass correlation coefficients (ICC), which can be used to assess the interobserver reproducibility of ROIs delineated, is obtained from the following equation:


$$ICC=\frac{\left(MS_{R}-MS_{E}\right)}{MS_{R}+\left(\frac{MS_{C}-MS_{E}}{n}\right)}$$

MSR: mean square for rows; MSC: mean square for columns; MSE: mean square for error; n: number of subjects.

After feature extraction, 80% of the dataset was randomly assigned to training set and for all cases, features were normalized to the normal distribution by mean and variance scaling. The Support Vector Machine (SVM) classifier was used to develop radiomic models based on single sequence and their combinations.

Since some of the extracted features can be invalid for the specific target task, it is necessary to identify features related to a specific task to achieve the optimal predictive performance. First, SelectKBest was applied to select the most significantly relevant feature set with threshold of 0.05. The least absolute shrinkage and selection operator (LASSO) is a regression analysis method that can perform both variable selection and regularization to improve the identification accuracy and interpretability of the model. For example, it has a tuning parameter to control the penalty of the linear model, which guarantees the minimum penalty when obtaining a model with a smaller number of features, where the penalty is mean square error (MSE). In addition, another parameter controls the correlation of features, making the selected features less relevant. L1 regularization was used as the cost function, the error value of cross validation was 5, and the maximum number of iterations was 1000. The optimization goal of LASSO is:


$$y=\left(\frac{1}{2*n_{samples}}\right)*{\left\|y-Xw\right\|}^{2}+alpha*\left\|w\right\|$$

where *X* is the radioactivity characteristic matrix, *y* is the sample vector marker, *n* is the sample number, *w* is the coefficient vector regression model, alpha∗∥*w*∥ is the LASSO punishment. A radiomic score for each patient was then computed using a linear combination of the key features weighted by their LASSO coefficients.

The SVM classifier was built to predict LVI based on final reduced radiomic features. The performance of the models was estimated by the receiver operating characteristic (ROC) curve and confusion matrix analysis with indicators of area under the curve (AUC), accuracy, sensitivity, and specificity.

### Statistical Analysis

Descriptive statistics and continuous variables were expressed as numbers (percentages) and mean (standard deviation, SD). Statistical analysis was performed using the Python 3.6 (https://www.python.org/). The univariate and multivariate logistic regression analysis was used to identify the independent clinical predictors of LVI status. The packages of “pyradiomics” (https://pyradiomics.readthedocs.io/), “scikitlearn” (https://scikit-learn.org/), and “matplotlib” (https://matplotlib. org/) were used for feature selection, model building, and plotting, respectively. A *P*-value <0.05 was considered statistically significant.

## Results

### Patient Characteristics and Clinical Model Construction

**Table 1** illustrates the clinical characteristics of patients in all datasets. The proportion of positive LVI was 32% (56/175). The patients from center 1 was randomly divided into a training set (n=99) and a validation set (n = 26), while the patients from center 2 was used as a test set (n=50).

**Table 1:** Demographic and clinical characteristics of patients.

Characteristics	Training dataset (n=99)	Validation dataset (n=26)	Test dataset (n=50)
Mean age (years)	45.8 ± 10.8	48.3 ± 9.3	53.4 ± 10.7
Tumor location			
Left	45 (45.5)	15 (57.7)	27 (54)
Right	54 (54.5)	11 (42.3)	23 (46)
Background parenchymal enhancement			
Minimal	19 (19.2)	7 (26.9)	21 (42)
Mild	32 (32.3)	8 (30.8)	16 (32)
Moderate	43 (43.4)	8 (30.8)	13 (26)
Marked	5 (5.1)	3 (11.5)	0
Fibroglandular tissue			
Almost entirely fat	6 (6.1)	3 (11.5)	1 (2)
Scattered fibroglandular tissue	33 (32.3)	11 (42.3)	5 (10)
Heterogeneous fibroglandular tissue	49 (49.5)	9 (34.7)	44 (88)
Extreme fibroglandular tissue	11 (11.1)	3 (11.5)	0
Chest wall invasion			
Yes	20 (20.2)	2 (7.8)	0
No	79 (79.8)	24 (92.2)	50 (100)
Pectoralis major muscle invasion			
Yes	11 (11.1)	2 (7.8)	1 (2)
No	88 (88.9)	24 (92.2)	49 (98)
Tumor diameter (mm)	34.9 ± 19.8	30.0 ± 11.4	23.7 ± 9.5
Mass shape			
Oval	2 (2)	0	14 (28)
Round	14 (14.1)	2 (7.8)	3 (6)
Irregular	83 (83.9)	24 (92.2)	33 (66)
Internal enhancement pattern			
Homogeneous	37 (37.4)	7 (26.9)	4 (8)
Heterogeneous	50 (50.5)	16 (61.5)	44 (88)
Rim enhancement	7 (7.1)	1 (3.8)	2 (4)
Dark internal septations	5 (5)	2 (7.8)	0
Tumor number			
Solitary	71 (71.7)	17 (65.4)	39 (78)
≥2	28 (28.3)	9 (34.6)	11 (22)
TNM stage			
I	14 (14.1)	3 (11.5)	14 (28)
II	51 (51.5)	16 (61.5)	28 (56)
III	26 (26.3)	6 (23.2)	7 (14)
IV	8 (8.1)	1 (3.8)	1 (2)
Pathological ALN status			
Absence	51 (51.5)	16 (61.5)	28 (56)
Single	5 (5)	3 (11.5)	5 (10)
≥2	43 (43.5)	7 (27)	17 (34)

The univariate logistic regression analysis showed that background parenchymal enhancement (*P* = 0.043), chest wall invasion (*P* = 0.022),axillary lymph node metastasis (P = 0.014),and pectoralis major muscle invasion (*P* = 0.021) remained as potential predictors of LVI status, however, they were nonsignificant (all *P* values >0.05) in the multivariate logistic regression analysis. The clinical model yielded an AUC of 0.74 (95%CI: 0.65–082), 0.61 (95%CI: 0.42–0.81), and 0.50 (95%CI: 0.40-0.66) in the training, validation, and test sets, respectively.

### Feature Extraction, Selection and Radiomic Signature Construction

Of all the radiomic features extracted, median ICC was 0.887, 871 (62%) features were robust, with ICC > 0.75. After using SelectKbest method and LASSO algorithm, 11, 2, 1, and 1 features were identified to develop the DCE, T2WI, DWI, and ADC based single-layered radiomic models (**Table 2**). **Table 3** shows the predictive performance of single-layered radiomic models and the fusion radiomic models. The results showed that ADC-based radiomic model achieved the optimal performance, with an AUC of 0.87 (95%CI: 0.80-0.94) in the training set and 0.87 (95%CI: 0.70-1.00) in the validation set. When validated in the test set, the ADC-based yielded an AUC of 0.77 (95%CI: 0.64-0.86).

**Table 2:** Radiomic features of the single-layered and fusion radiomic models.

Models	Features	Number
DCE	original_shape_Maximum2DDiameterColumn original_glcm_Idmn logarithm_glszm_HighGrayLevelZoneEmphasis wavelet-LHL_glcm_Imc2 wavelet-LHH_glcm_MaximumProbability wavelet-LLH_glszm_GrayLevelVariance wavelet-LLH_glszm_LowGrayLevelZoneEmphasis wavelet-LLH_glszm_ZoneEntropy wavelet-HLH_glszm_GrayLevelNonUniformityNormalized wavelet-HHL_glrlm_HighGrayLevelRunEmphasis wavelet-LLL_glszm_SmallAreaHighGrayLevelEmphasis	11
T2WI	wavelet-HHH_glszm_SmallAreaLowGrayLevelEmphasis wavelet-HLH_glrlm_ShortRunEmphasis	2
DWI	wavelet-HHL_glszm_SmallAreaLowGrayLevelEmphasis	1
ADC	wavelet-HLH_gldm_GrayLevelVariance	1
DCE+T2WI	DCE_original_shape_Maximum2DDiameterColumn DCE_exponential_glszm_GrayLevelNonUniformity DCE_gradient_glszm_ZoneEntropy DCE_wavelet-LHH_glcm_JointEnergy DCE_wavelet-HLH_glszm_GrayLevelNonUniformityNormalized T2_wavelet-LHH_glszm_SizeZoneNonUniformityNormalized T2_wavelet-LLH_glszm_GrayLevelVariance T2_wavelet-LLH_glszm_SmallAreaEmphasis T2_wavelet-HLH_glrlm_ShortRunHighGrayLevelEmphasis T2_wavelet-HLH_glszm_GrayLevelVariance T2_wavelet-HHH_glszm_SmallAreaLowGrayLevelEmphasis T2_wavelet-LLL_ngtdm_Strength	12
DCE+DWI	DWI_wavelet-HHL_glszm_SizeZoneNonUniformityNormalized DWI_wavelet-HHL_glszm_SmallAreaLowGrayLevelEmphasis	2
DCE+ADC	DCE_original_shape_Maximum2DDiameterColumn DCE_wavelet-HLL_glrlm_LongRunLowGrayLevelEmphasis DCE_wavelet-HLH_glszm_GrayLevelNonUniformityNormalized ADC_exponential_gldm_DependenceEntropy ADC_wavelet-LHL_firstorder_Maximum ADC_wavelet-LHL_firstorder_RootMeanSquared ADC_wavelet-LLH_glrlm_ShortRunEmphasis	7
T2WI+DWI	DWI_wavelet-LLH_gldm_DependenceVariance DWI_wavelet-HHL_glszm_SizeZoneNonUniformityNormalized T2_wavelet-LHH_glszm_SizeZoneNonUniformityNormalized T2_wavelet-LLH_glszm_SmallAreaEmphasis T2_wavelet-HHH_glrlm_LowGrayLevelRunEmphasis T2_wavelet-HHH_glszm_SmallAreaLowGrayLevelEmphasis T2_wavelet-LLL_glrlm_ShortRunLowGrayLevelEmphasis T2_wavelet-LLL_glrlm_LongRunLowGrayLevelEmphasis	8
T2WI+ADC	T2_wavelet-LLH_glszm_SmallAreaHighGrayLevelEmphasis T2_wavelet-LLH_glszm_SmallAreaEmphasis ADC_wavelet-HHH_glrlm_ShortRunEmphasis	3
DWI+ADC	ADC_wavelet-LLH_glrlm_ShortRunEmphasis	1
DCE+T2WI+DWI	DCE_wavelet-HHH_glszm_ZoneEntropy DWI_wavelet-HHL_glszm_SmallAreaLowGrayLevelEmphasis T2_wavelet-LLH_glszm_SmallAreaEmphasis	3
DCE+T2WI+ADC	T2_wavelet-LHL_firstorder_Skewness T2_wavelet-LLH_glszm_SmallAreaEmphasis T2_wavelet-HHH_glrlm_LowGrayLevelRunEmphasis T2_wavelet-HHH_glszm_HighGrayLevelZoneEmphasis ADC_exponential_gldm_DependenceEntropy ADC_wavelet-LHL_firstorder_Maximum ADC_wavelet-LHL_firstorder_RootMeanSquared ADC_wavelet-HLL_firstorder_Maximum DCE_original_shape_Maximum2DDiameterColumn DCE_wavelet-HLL_glrlm_LongRunLowGrayLevelEmphasis DCE_wavelet-LLH_glszm_GrayLevelVariance DCE_wavelet-HLH_glszm_GrayLevelNonUniformityNormalized	12
DCE+DWI+ADC	ADC_wavelet-LLH_glrlm_ShortRunEmphasis	1
T2WI+DWI+ADC	ADC_wavelet-LLH_glrlm_ShortRunEmphasis	1
DCE+T2WI+DWI+ADC	ADC_wavelet-LLH_glrlm_ShortRunEmphasis	1

**Table 3:** Predictive performance of single-layered and fusion radiomic models.

Models	Sensitivity			Specificity			AUC (95%CI)		
	Training set	Validation set	Test set	Training set	Validation set	Test set	Training set	Validation set	Test set
ADC	0.83	0.63	0.63 0.13	0.85	1.00	0.73 0.91	0.87 (0.80-0.94)	0.87 (0.7–1.00)	0.77 (0.64-0.86) 0.82 (0.64,1.00)
DWI	0.77	0.64	0.60 0.25	0.53	0.60	0.64 0.91	0.68 (0.59-0.76)	0.64 (0.42-0.83)	0.58 (0.47-0.70) 0.74 (0.51-0.92)
T2WI	0.67	0.75	0.60 0.25	1.00	0.73	0.63 0.91	0.89 (082-0.95)	0.64 (0.42-0.83)	0.58 (0.50-0.71) 0.70 (0.48,0.89)
DCE	0.88	0.64	0.75 0.50	0.76	0.63	0.69 0.73	0.88 (0.82-0.93)	0.68 (0.50-0.86)	0.64 (0.51-0.80) 0.65 (0.41-0.87)
DCE+T2WI	0.93	0.64	0.65 0.50	0.74	0.74	0.74 0.91	0.90 (0.84-0.95)	0.68 (0.48-0.88)	0.62 (0.49-0.76) 0.58 (0.35-0.83)
DCE+DWI	0.83	0.88	0.60 0.12	0.61	0.65	0.67	0.76 (0.66-0.85)	0.64 (0.40-0.87)	0.61 (0.48-0.80) 0.68 (0.44-0.89)
DCE+ADC	0.71	0.63	0.60 0.38	0,85	0.93	0.71 0.55	0.85 (0.78-0.90)	0.70 (0.50-0.88)	0.62 (0.52-0.75) 0.53 (0.30-0.77)
T2WI+DWI	1.00	0.65	0.75	0.98	0.93	0.61 0.45	0.99 (0.97-1.00)	0.70 (0.48-0.88)	0.59 (0.51-0.70) 0.70 (0.48-0.91)
T2WI+ADC	0.63	0.64	0.70 0.38	0.76	0.67	0.65 0.82	0.74 (0.66-0.82)	0.65 (0.46-0.83)	0.60 (0.46-0.76) 0.56 (0.28-0.81)
DWI+ADC	0.60	0.63	0.65 0.50	0.76	0.67	0.66 0.55	0.66 (0.57-0.75)	0.70 (0.51-0.88)	0.65 (0.53-0.80) 0.53 (0.29-0.77)
DCE+T2WI+DWI	0.93	0.82	0.63 0.88	0.83	0.61	0.67 0.45	0.91 (0.66-0.85)	0.73 (0.40-0.87)	0.62 (0.40-0.79) 0.64 (0.40-0.87)
DCE+T2WI+ADC	0.90	0.62	0.75 0.13	0.88	0.80	0.69 0.91	0.93 (0.89-0.97)	0.64 (0.44-0.82)	0.58 (0.45-0.75) 0.66 (0.39-0.90)
DCE+DWI+ADC	0.68	0.63	0.75 0.50	0.78	0.70	0.68 0.73	0.78 (0.70-0.86)	0.62 (0.42-0.81)	0.53 (0.44-0.67) 0.56 (0.30-0.81)
T2WI+DWI+ADC	0.60	0.67	0.63 0.38	0.76	0.68	0.73 0.82	0.66 (0.57-0.75)	0.70 (0.51-0.88)	0.69 (0.47-0.89) 0.73 (0.50-0.93)
DCE+T2WI+DWI+ADC	0.68	0.65	0.63 0.25	0.78	0.73	0.67 0.91	0.78(0.70-0.86)	0.62 (0.42-0.81)	0.66 (0.43-0.90) 0.68 (0.44-0.89)

## Discussion

Breast cancer with LVI is the pathological manifestation of tumor emboli in the lymphatic and blood vessels in the vicinity of invasive breast cancer. The presence of LVI increases the risk of axillary lymph node metastasis and distant metastasis and is associated with a poor prognosis (5). Currently, LVI can only be confirmed *via* the pathological assessment of specimens after resection. The building of radiomic model allows preoperative evaluation of LVI status. It is of great clinical significance as the presence or absence of LVI is a crucial criterion for treatment planning.

MRI has been used as one of the preferred imaging methods for early screening of breast cancer, assessment of malignancy, and determination of efficacy and prognosis. Previous studies suggested that some MRI features were significantly associated with LVI status, such as background parenchymal enhancement, peritumoral edema, adjacent vessel sign, enhancement types, and MRI-reported axillary lymph node metastasis (14, 16–18). However, these features were somewhat subjective and could be affected by sample size of a study. In this current study, we observed no any clinical variables included were independent risk factors of LVI, possibly due to small sample size of our training set. More objective and reliable markers are desirable to identification of LVI status in patients with breast cancer.

Radiomics uses high-throughput extraction of high-level quantitative features to describe tumor phenotypes objectively and quantitatively. These features are extracted from medical imaging data using advanced mathematical algorithms, revealing tumor features that may not be discernible with the naked eye (19). Radiomics may have great potential in capturing important phenotypic data on tumors, such as intratumoral heterogeneity (20, 21), thus providing valuable information for individualized clinical treatment. The use of radiomic approach to determine the prognostic factors of breast cancer is almost completely dominated by DCE-MRI, which provides not only abundant radiomic data but also functional information reflecting the DCE parameter characteristics of the tumor. Kinetic enhancement curve was identified as a predictor of LVI (22). Liu et al. showed that DCE-based radiomics signature in combination with MRI ALN status was effective in predicting the LVI status, with an AUC of 0.763 (12); however, this study was limited due to lack of external validation and comparison with other MRI sequences. T2WI allows clear delineation of the lesions, high contrast of the surrounding soft tissue, clear depiction of the size and shape of the lesion, and greater sensitivity to cystic changes and necrosis within the lesion. Adding radiomic features extracted from T2WI images may improve the diagnostic performance of other MRI sequences (14). DWI is a functional imaging method that reflects the Brownian motion of water molecules in the body. ADC value is a quantitative indicator associated with the diffusion of water molecules and microcirculatory perfusion. Previous studies have demonstrated that tumor and peritumoral ADC values were significantly correlated with LVI status (23, 24). The quantitative ADC obtained from DWI has been increasingly used to improve the diagnostic accuracy of contrast-enhanced MRI in breast cancer (14). The results of this present study were consistent with a recent study by Kayadibi et al. (13). in which the ADC-based radiomic model could predict LVI status with satisfying performance. Zhang et al. (14) found that the fusion radiomic signature of the T2WI, cT1WI, and ADC maps achieved a better predictive efficacy for LVI than either of them alone, which was inconsistent with our study that reported the combination of multiparametric MRI-derived radiomic features failed to achieve a complementary effect in the prediction of LVI status. Thus, the role of fusion radiomic model needs to be tested in larger datasets.

The limitations of our study need to be acknowledged. First, this was a retrospective study with small sample size, a multicenter study with a larger sample size is warranted. Second, LVI status was only classified as positive or negative in this study. Uematsu et al. (25), divided into four grades according to the number of lymphovascular structures invaded. Further studies should evaluate the association between radiomic features with different grades of LVI. Third, the influence of MRI parameters on the radiomic features was not analyzed due to the small sample size. Finally, only radiomic features derived from the first postcontrast images of DCE-MRI were analyzed due to its crucial role in the diagnostic performance of breast MRI. The precontrast, other DCE-MRI series deserve to be investigated in further studies.

## Conclusion

Our results showed that radiomic features based on ADC map could be used to effectively predict LVI status in invasive breast cancer, potentially improving preoperative diagnosis and patient-specific treatment planning. However, the findings of this preliminary study needs to be validated in larger datasets.

## Data Availability Statement

The original contributions presented in the study are included in the article/supplementary material. Further inquiries can be directed to the corresponding author.

## Ethics Statement

The studies involving human participants were reviewed and approved by The First People’s Hospital of Kashgar. The ethics committee waived the requirement of written informed consent for participation.

## Author Contributions

MN, DA, and GD: conception and design. AH: provision of study materials or patients. AA, SM, PM, and NR: collection and assembly of data. MN, AH, and AA: data analysis and interpretation. All authors: manuscript writing and final approval of the manuscript.

## Funding

This work was supported by the Special Scientific Research Project of Health Young Medical Science and Technology Talents in Xinjiang Uygur Autonomous Region (Grant No. WJWY202103).

## Conflict of Interest

The authors declare that the research was conducted in the absence of any commercial or financial relationships that could be construed as a potential conflict of interest.

## Publisher’s Note

All claims expressed in this article are solely those of the authors and do not necessarily represent those of their affiliated organizations, or those of the publisher, the editors and the reviewers. Any product that may be evaluated in this article, or claim that may be made by its manufacturer, is not guaranteed or endorsed by the publisher.

## References

[1] Bray F Ferlay J Soerjomataram I Siegel RL Torre LA Jemal A. Global Cancer Statistics 2018: GLOBOCAN Estimates of Incidence and Mortality Worldwide for 36 Cancers in 185 Countries. CA Cancer J Clin (2018) 68:394–424. 30207593 10.3322/caac.21492

[2] Siegel RL Miller KD Jemal A. Cancer Statistics, 2016. Cancer J Clin (2016) 66:7–30. 10.3322/caac.21332 26742998

[3] Huang KT Kim YA Kim J Chu AJ Chang JH Oh SW. The Influences of Peritumoral Lymphatic Invasion and Vascular Invasion on the Survival and Recurrence According to the Molecular Subtypes of Breast Cancer. Breast Cancer Res Treat (2017) 163:71–82. 28194538 10.1007/s10549-017-4153-4

[4] Yi M Mittendorf EA Cormier JN Buchholz TA Bilimoria K Sahin AA. Novel Staging System for Predicting Disease-Specific Survival in Patients With Breast Cancer Treated With Surgery as the First Intervention: Time to Modify the Current American Joint Committee on Cancer Staging System. J Clin Oncol (2011) 29:4654–61. 10.1200/JCO.2011.38.3174 PMC3236648 22084362

[5] Ejlertsen B Jensen MB Rank F Rasmussen BB Christiansen P Kroman N. Population-Based Study of Peritumoral Lymphovascular Invasion and Outcome Among Patients With Operable Breast Cancer. J Natl Cancer Inst (2009) 101:729–35. 10.1093/jnci/djp090 19436035

[6] Schoppmann SF Bayer G Aumayr K Taucher S Geleff S Rudas M. Prognostic Value of Lymphangiogenesis and Lymphovascular Invasion in Invasive Breast Cancer. Ann Surg (2004) 240:306–12. 10.1097/01.sla.0000133355.48672.22 PMC1356408 15273556

[7] Viale G Giobbie-Hurder A Gusterson BA Maiorano E Mastropasqua MG Sonzogni A. Adverse Prognostic Value of Peritumoral Vascular Invasion: Is it Abrogated by Adequate Endocrine Adjuvant Therapy? Results From Two International Breast Cancer Study Group Randomized Trials of Chemoendocrine Adjuvant Therapy for Early Breast Cancer. Ann Oncol (2010) 21:245–54. 10.1093/annonc/mdp317 PMC2813305 19633051

[8] Davis BW Gelber R Goldhirsch A Hartmann WH Hollaway L Russell I. Prognostic Significance of Peritumoral Vessel Invasion in Clinical Trials of Adjuvant Therapy for Breast Cancer With Axillary Lymph Node Metastasis. Hum Pathol (1985) 16:1212–8. 10.1016/s0046-8177(85)80033-2 3905576

[9] Colleoni M Rotmensz N Maisonneuve P Sonzogni A Pruneri G Casadio C. Prognostic Role of the Extent of Peritumoral Vascular Invasion in Operable Breast Cancer. Ann Oncol (2007) 18:1632–40. 10.1093/annonc/mdm268 17716986

[10] Bera K Braman N Gupta A Velcheti V Madabhushi A. Predicting Cancer Outcomes With Radiomics and Artificial Intelligence in Radiology. Nat Rev Clin Oncol (2022) 19:132–46. 10.1038/s41571-021-00560-7 PMC9034765 34663898

[11] Lambin P Leijenaar RTH Deist TM Peerlings J de Jong EEC van Timmeren J. Radiomics: The Bridge Between Medical Imaging and Personalized Medicine. Nat Rev Clin Oncol (2017) 14:749–62. 10.1038/nrclinonc.2017.141 28975929

[12] Liu Z Feng B Li C Chen Y Chen Q Li X. Preoperative Prediction of Lymphovascular Invasion in Invasive Breast Cancer With Dynamic Contrast-Enhanced-MRI-Based Radiomics. J Magn Reson Imaging (2019) 50:847–57. 10.1002/jmri.26688 30773770

[13] Kayadibi Y Kocak B Ucar N Akan YN Yildirim E Bektas S. MRI Radiomics of Breast Cancer: Machine Learning-Based Prediction of Lymphovascular Invasion Status. Acad Radiol (2022) null:S126–34. 10.1016/j.acra.2021.10.026 34876340

[14] Zhang J Wang G Ren J Yang Z Li D Cui Y. Multiparametric MRI-Based Radiomics Nomogram for Preoperative Prediction of Lymphovascular Invasion and Clinical Outcomes in Patients With Breast Invasive Ductal Carcinoma. Eur Radiol (2022) 36(6):4079–89. doi: 10.1007/s00330-021-08504-6 35050415

[15] Magny SJ Shikhman R Keppke AL. Breast Imaging Reporting and Datasystem, ACR BI-RADS. In: American College of Radiology, 4th ed. Reston, VA: StatPearls Publishing, Treasure Island (FL) (2003).

[16] Ni-Jia-Ti MY Ai-Hai-Ti DL Huo-Jia AS Wu-Mai-Er PL A-Bu-Li-Zi AB Shi Y. Development of a Risk-Stratification Scoring System for Predicting Lymphovascular Invasion in Breast Cancer. BMC Cancer (2020) 20:94. 32013960 10.1186/s12885-020-6578-0 PMC6998851

[17] Li J Mo Y He B Gao Q Luo C Peng C. Association Between MRI Background Parenchymal Enhancement and Lymphovascular Invasion and Estrogen Receptor Status in Invasive Breast Cancer. Br J Radiol (2019) 92:20190417. 31398071 10.1259/bjr.20190417 PMC6849688

[18] Koh J Park AY Ko KH Jung HK. Can Enhancement Types on Preoperative MRI Reflect Prognostic Factors and Surgical Outcomes in Invasive Breast Cancer? Eur Radiol (2019) 29:7000–8. 10.1007/s00330-019-06236-2 31187220

[19] Cheon H Kim HJ Lee SM Cho SH Shin KM Kim GC. Preoperative MRI Features Associated With Lymphovascular Invasion in Node-Negative Invasive Breast Cancer: A Propensity-Matched Analysis. J Magn Reson Imaging (2017) 46:1037–44. 10.1002/jmri.25710 28370761

[20] Lim Y Ko ES Han BK Ko EY Choi JS Lee JE. Background Parenchymal Enhancement on Breast MRI: Association With Recurrence-Free Survival in Patients With Newly Diagnosed Invasive Breast Cancer. Breast Cancer Res Treat (2017) 163:573–86. 10.1007/s10549-017-4217-5 28349273

[21] Gillies RJ Kinahan PE Hricak H. Radiomics: Images Are More Than Pictures, They are Data. Radiology (2016) 278:563–77. 10.1148/radiol.2015151169 PMC4734157 26579733

[22] Grossmann P Stringfield O El-Hachem N Bui MM Rios Velazquez E Parmar C. Defining the Biological Basis of Radiomic Phenotypes in Lung Cancer. Elife (2017) 6:e23421. 28731408 10.7554/eLife.23421 PMC5590809

[23] Guo Y Hu Y Qiao M Wang Y Yu J Li J. Radiomics Analysis on Ultrasound for Prediction of Biologic Behavior in Breast Invasive Ductal Carcinoma. Clin Breast Cancer (2018) 18:e335–44. 10.1016/j.clbc.2017.08.002 28890183

[24] Choi BB. Dynamic Contrast Enhanced-MRI and Diffusion-Weighted Image as Predictors of Lymphovascular Invasion in Node-Negative Invasive Breast Cancer. World J Surg Oncol (2021) 19:76. 33722246 10.1186/s12957-021-02189-3 PMC7962354

[25] Mori N Mugikura S Takasawa C Miyashita M Shimauchi A Ota H. Peritumoral Apparent Diffusion Coefficients for Prediction of Lymphovascular Invasion in Clinically Node-Negative Invasive Breast Cancer. Eur Radiol (2016) 26:331–9. 10.1007/s00330-015-3847-4 26024846

